# Weight loss increases circadian gene expression and emotional well-being in individuals with obesity

**DOI:** 10.3389/fnut.2025.1722428

**Published:** 2025-11-26

**Authors:** Carmen Grau-del Valle, Neus Bosch-Sierra, Alberto Hermo-Argibay, Sandra López-Domenech, Milagros Rocha, Víctor M. Víctor, Carlos Morillas, Susana Rovira-Llopis, Celia Bañuls

**Affiliations:** 1 Department of Endocrinology and Nutrition, University Hospital Doctor Peset, Foundation for the Promotion of Health and Biomedical Research in the Valencian Region (FISABIO), Valencia, Spain; 2 National Network of Biomedical Research on Hepatic and Digestive Diseases (CIBEREHD), Madrid, Spain; 3 Department of Physiology, University of Valencia, INCLIVA (Biomedical Research Institute Valencia), Valencia, Spain; 4 Faculty of Medicine and Odontology, Department of Medicine, University of Valencia, Valencia, Spain

**Keywords:** obesity, weight loss, circadian rhythm, self-esteem, depression, anxiety, clock genes

## Abstract

**Introduction:**

Obesity is associated with disruptions in circadian rhythms and emotional well-being that can contribute to metabolic and psychological health issues. This study aimed to investigate the effects of weight loss by a dietary intervention on circadian gene expression and emotional state in individuals with obesity.

**Methods:**

Fifty subjects with obesity followed a dietary intervention for 6 months. Anthropometric and biochemical parameters were assessed. Sleep quality was measured using a sleep questionnaire and Rosemberg Self-Esteem Scale, Beck Depression Inventory and State-Trait Anxiety Inventory (STAI) were used. PBMCs’ RNA was analyzed for circadian-related gene expression.

**Results:**

Participants lost 11.3% body weight, along with reductions in lipid, carbohydrate metabolism and inflammation markers. Sleep quality improved; as poor sleep frequency was reduced, though sleep duration was similar. Self-esteem did not significantly increase; however, the proportion of participants with high self-esteem rose from 62 to 69% (*χ*^2^ = 12.5, *p* < 0.05). State anxiety decreased (STAI-S, *p* < 0.01), though trait anxiety remained stable. Several circadian genes were upregulated, including *CLOCK*, *ARNTL*, *CRY1*, *DBP*, and *NR1D1*; with associations found between *CLOCK* and lower state anxiety at baseline, and between *CRY2* and higher self-esteem and lower depression at baseline (*p* < 0.05).

**Conclusion:**

The dietary intervention influenced positively the metabolic, psychological, and circadian rhythm marker profile, highlighting potential interconnections between metabolism, circadian gene expression, and mental well-being.

**Clinical Trial Registration:**

Identifier NCT06279780

## Graphical abstract

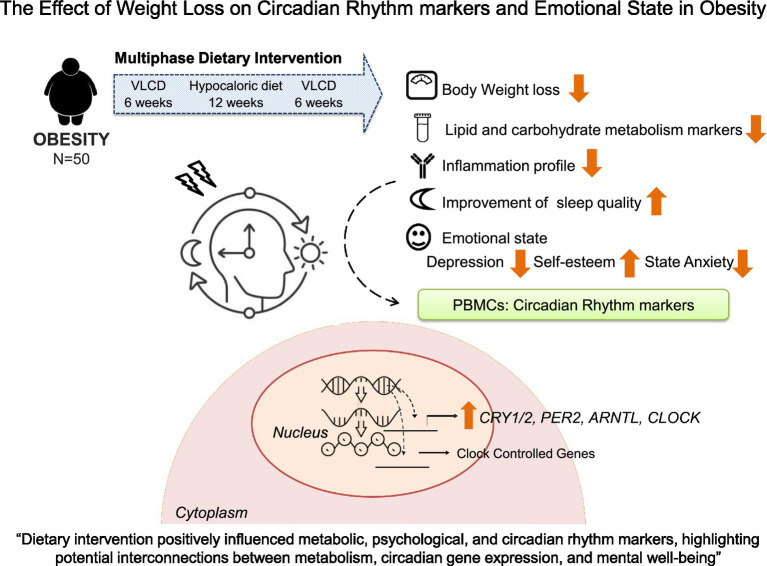

## 1 Introduction

Obesity is a global public health issue characterized by excessive fat accumulation, and which presents significant risks to physical and mental well-being. Individuals with obesity often experience a range of health complications, including cardiovascular diseases, metabolic and psychological disorders (1), which affect their daily living patterns and increase the global burden of the disease (2). In this context, recent studies have shown that sleep disturbance and altered circadian rhythms – natural 24-h cycles that regulate sleep, feeding, and other physiological processes – contribute to the development and maintenance of obesity, and they also relate to mental health problems such as depression and anxiety (3). In fact, difficulties in falling asleep and maintaining an adequate circadian rhythm can be both cause and consequence of these psychological disorders (4).

From a physiological perspective, circadian cycles in the body are regulated by the central clock located at the suprachiasmatic nucleus (SCN) of the hypothalamus and peripheral clocks residing in a multitude of tissues throughout the body (5). The molecular circadian clock involves a transcription–translation feedback loop composed of several “core” proteins, including Brain and muscle Arnt-like protein-1 (BMAL1) (encoded by *ARNTL*), Circadian Locomotor Output Cycles Kaput (CLOCK), Cryptochrome Circadian Regulator (CRY), and Period Circadian Regulator (PER) (6). Other key circadian regulators include nuclear receptor subfamily 1 group D member 1 (REV-ERBα; encoded by *NR1D1*), which inhibits BMAL1 expression, and D-box Binding Protein (*DBP*) and Krüppel-like factor 9 (*KLF9*), which are circadian clock genes under CLOCK-BMAL1 transcriptional regulation. In addition, Basic Helix–Loop–Helix Family Member e41 (BHLHE41) transcription factor and Circadian Associated Repressor of Transcription (CIART) contribute to the fine regulation of the circadian system by suppressing CLOCK-BMAL-induced transactivation (7, 8).

The central circadian clock regulates food intake, energy expenditure, and insulin sensitivity, while peripheral clocks fine-tune these processes within metabolic tissues. In individuals with obesity, circadian disruption often resulting from irregular sleep patterns, excessive caloric intake, and sedentary lifestyles can aggravate metabolic dysregulation, establishing a feedback loop that promotes further weight gain (4, 9). Moreover, circadian misalignment has been linked to impaired mood regulation and increased susceptibility to stress, depression, and anxiety (10, 11). Thus, obesity contributes, at least partially, to both metabolic and psychological disturbances through circadian disruption.

Importantly, weight loss through lifestyle changes, such as diet and exercise, has been associated with improvements in sleep quality, circadian rhythm regulation, and hormonal balance, as well as an improvement in symptoms of depression and anxiety. These findings highlight the relevance of understanding the interconnectedness of weight, circadian rhythms, and mental health when developing effective interventions for obesity (12, 13).

Therefore, the aim of the present study was to evaluate how a six-month dietary weight loss intervention could influence levels of self-esteem, depression, and anxiety, and to explore correlations with gene expression of peripheral markers related to circadian rhythm in obese individuals.

## 2 Materials and methods

### 2.1 Subjects

A total of 50 individuals with obesity were selected from participants seeking weight loss treatment at the Endocrinology and Nutrition Department of University Hospital Dr. Peset (Valencia, Spain). This subgroup was selected from a previously described cohort (14), based on the availability of complete psychological questionnaire data and peripheral blood mononuclear cell (PBMC) samples for gene expression analyses. To be eligible, participants had to be between 18 and 60 years old and to have a body mass index (BMI) of at least 30 kg/m^2^, to have a confirmed obesity diagnosis for at least 5 years and to have maintained a stable weight during the 3 months prior to the study. Metabolic syndrome was defined according to the National Cholesterol Education Program (NCEP) Adult Treatment Panel III (ATP III) criteria. Participants’ medical records were reviewed for a prior diagnosis of sleep apnea confirmed by a pulmonologist. Those who had a positive STOP-Bang screening or a previous diagnosis were referred for reassessment by a pulmonologist after the intervention.

Exclusion criteria included any serious illness, a history of chronic inflammatory conditions, secondary obesity (such as untreated hypothyroidism or Cushing’s syndrome), pregnancy, breastfeeding and reading comprehension difficulties.

The Ethics Committee of the hospital approved the study (Code: 92/18), which was conducted in accordance with the Declaration of Helsinki guidelines. All participants provided written informed consent.

### 2.2 Dietary intervention

After the initial assessment, patients completed 26-week cycles of a very low-calorie diet (VLCD) using a liquid formula (Optisource, Nestlé S.A., Vevey, Switzerland) providing 82.2 g carbohydrates, 45.0 g protein, 13.5 g fat, minimal fiber, essential vitamins/minerals, and 2,658 kJ/day (630 kcal/day).

Between cycles, they followed a 12-week personalized hypocaloric diet, reducing ~500 kcal/day from estimated resting energy expenditure. Diets were adapted to individual habits and macronutrient guidelines (50%–55% carbohydrates, 15% protein, 30%–35% fat), emphasized fiber-rich foods (fruits, legumes, vegetables, whole grains), and limited added sugars.

Dietary adherence was monitored every 6 weeks through structured interviews and dietary records, while clinical dietitian and endocrinologists supervised VLCD cycles to minimize nutritional bias and prevent adverse effects (e.g., dizziness, fatigue, micronutrient deficiencies). Participants also received detailed instructions and individualized counseling to promote adherence, and were advised to drink >2 L calorie-free fluids daily, and maintained their usual medical prescriptions. The weight loss goal was defined as a 10% reduction from the initial body weight.

### 2.3 Psychological instruments

Participants completed paper-based questionnaires assessing sleep quality, self-esteem, depression, and anxiety before and after the dietary intervention.

#### 2.3.1 Sleep quality

A brief, non-validated set of sleep-related questions was administered to assess subject’s sleep quality. The items were focused on estimating the total number of hours sleep per night and the frequency of nights of poor sleep during the preceding week. Depending on the item, responses were captured using a 5-point Likert scale, as multiple-choice questions with defined options, or open-ended numerical values.

#### 2.3.2 Self-esteem

Self-esteem was measured using the Rosenberg Self-Esteem Scale, which is indicated when exploring feelings of personal worth and self-respect. The scale consisted of 10 items, of which five were stated positively and five negatively to control for the effect of acquiescence. A 4-point Likert-type scale was used, offering four response options: 1 (strongly disagree), 2 (disagree), 3 (agree), and 4 (strongly agree). Total scores ranged from 10 to 40, with higher scores indicating higher self-esteem, according to the following categories: low self-esteem (scores below 25), moderate self-esteem (scores 26–29) and high self-esteem (scores 30–40).

#### 2.3.3 Depression

Depression was assessed using the Beck Depression Inventory-II (BDI-II), a 21-item self-report measure that evaluates symptoms such as sadness, crying, loss of pleasure, feelings of failure and guilt, suicidal thoughts or wishes and pessimism. Each item was answered on a 4-point scale, from 0 (no symptoms) to 3 (severe symptoms), except for items 16 (changes in sleep pattern) and 18 (changes in appetite), which contain 7 categories. The minimum and maximum scores are 0 and 63. Depression levels were categorized as follows: minimal depression: 0–13; mild depression: 14–19; moderate depression: 20–28; severe depression: 29–63.

#### 2.3.4 State–trait anxiety

Anxiety was evaluated using the State-Trait Anxiety Inventory (STAI), which consisted of two subscales that measure state anxiety and trait anxiety. The State Anxiety subscale consisted of 20 items which assess a transitory emotional state. Subjects rated how they feel “right now” on a 4-point scale (1 = not at all, 4 = very much so). The Trait Anxiety subscale (STAI-T) also consisted of 20 items, and pointed to a relatively stable anxious propensity that characterized individuals with a tendency to perceive situations as threatening. Participants rated how they “generally feel” on a 4-point scale (1 = almost never, 4 = almost always). Scores for both subscales ranged from 20 to 80, with higher scores indicating higher levels of anxiety. Anxiety severity was categorized into five levels: very low anxiety: 20–30; low anxiety: 31–40; moderate anxiety: 41–50; high anxiety: 51–60 and very high anxiety: 61–80.

### 2.4 Body composition and biochemical determinations

Anthropometric measurements, as well as systolic and diastolic blood pressure (BP) and body composition (assessed via bioelectrical impedance), were evaluated at baseline and post-intervention.

Blood samples were collected in the morning (between 8.00 and 9.00 a.m.) after a 12-h fasting period, both at baseline and post-intervention. The following variables were measured: glucose and lipid levels, liver and kidney function, nutritional status, hormone levels, complete blood count, and coagulation factors. All analyses were carried out by the hospital’s Clinical Analysis Department.

Plasminogen activator inhibitor 1 (PAI-1) and adipokine levels (adiponectin) were analyzed with the Luminex® 200 system (Luminex Corporation, Austin, TX, USA), following the procedure outlined by the MILLIPLEX® kit manufacturer (Millipore Corporation, Billerca, MA, USA).

### 2.5 RNA extraction and RT-qPCR

RNA was isolated from participants’ PBMCs (2.5 × 10^6^ cells) using the Ribospin RNA Extraction Kit (GeneAll, Seoul, Korea) and stored at −80 °C in RNA (Thermo Fisher Scientific, Waltham, MA, USA). RNA concentration and purity were measured with a Nanodrop 2000 (Thermo Fisher Scientific), targeting an A260/A280 ratio near 2. A total of 1,000 ng RNA was reverse transcribed into cDNA with the RevertAid First Strand cDNA Synthesis Kit (Thermo Fisher Scientific) under the following conditions: 5 min at 25 °C, 60 min at 42 °C, 5 min at 70 °C, then cooling at 4 °C, yielding 20 μL.

Target genes *CLOCK, ARNTL, CRY1, CRY2, PER1, DBP, BHLHE41, NR1D1, KLF9*, and *18S* were amplified and quantified using the 7500 Fast Real-Time PCR System (Thermo Fisher Scientific) and FastStart Universal SYBR Green (Sigma-Aldrich, St. Louis, MO, USA). Primers were designed with NCBI primer-BLAST (sequences in Supplementary Table 1).

PCR conditions: 10 min at 95 °C, 40 cycles of 10 s at 95 °C and 30 s at 60 °C, followed by melting curve analysis (2 cycles: 15 s at 95 °C, 1 min at 60 °C). Final reaction volume was 10 μL. All samples were run in duplicate, normalized to 18S RNA, and expressed as ΔΔCt. RNAseAway (Thermo Fisher Scientific) was used throughout, and no-template controls were included.

### 2.6 Statistical analysis

For the statistical analysis, we used SPSS software version 22.0 (SPSS Statistics IMC, Chicago, IL, USA). Continuous variables are reported as mean ± standard deviation (SD) for parametric data or as median and interquartile range (25th–75th percentile) for non-parametric data. Categorical variables are displayed as percentages. For comparisons, we applied either a paired Student *t*-test for parametric data or a Wilcoxon test for non-parametric data. Correlations between variables were analyzed using Spearman’s Rho bivariate correlation. All tests were conducted with a 95% confidence interval (CI), and *p*-values below 0.05 were considered statistically significant.

## 3 Results

This study included 50 individuals, 66% of whom were female, with an average age of 43.2 ± 10.0 years and a mean BMI of 42.0 ± 8.4 kg/m^2^. A total of 35% had been diagnosed with hypertension, 6% with type 2 diabetes, and 20% had dyslipidemia. At baseline, metabolic syndrome was confirmed in 58% of the participants and 22% presented sleep apnea. Additionally, three participants reported daily pharmacological treatment with anxiolytic medication, which had been maintained stable for at least 3 months prior to study inclusion.

Following the dietary intervention, 72% successfully achieved the weight loss goal. Specifically, subjects experienced an average weight reduction of 11.1 ± 8.1%, accompanied by significant reductions in fat mass (10.7 ± 11.1 Kg) and visceral fat volume (1.7 ± 1.6 L). Furthermore, a substantial decrease was observed in both systolic and diastolic BP, along with notable improvements in lipid (total cholesterol; TC and triglycerides; TG) and carbohydrate metabolism (glucose, insulin, HOMA-IR, hemoglobin A1c) and adiponectin (Table 1). Moreover, there were reductions in several inflammatory markers, such as acid uric, high-sensitivy c-reactive protein (hs-CRP), C3 protein (C3), neutrophil and monocyte count, and plasminogen activator inhibitor 1 (PAI-1), indicating an overall amelioration of the subjects’ inflammatory and metabolic profile (Table 2).

**Table 1:** Anthropometric and metabolic outcomes in the study population.

Parameter	Baseline	Final
*N* (females %)	50 (66.0)	—
Age (years)	43.2 ± 10.0	—
Weight (kg)	115.9 ± 23.9	102.8 ± 22.1***
BMI (kg/m^2^)	42.0 ± 8.4	37.3 ± 7.9***
Waist (cm)	120.0 ± 17.8	108.3 ± 17.9***
Fat mass index (kg/m^2^)	20.2 ± 6.2	16.8 ± 6.0***
Fat-free mass index (kg/m^2^)	21.4 ± 3.1	20.3 ± 3.1 ***
Visceral fat (L)	5.7 ± 3.4	4.0 ± 2.8***
Systolic BP (mmHg)	131.4 ± 18.2	119.8 ± 25.1**
Diastolic BP (mmHg)	79.6 ± 10.6	75.7 ± 10.5*
Glucose (mg/dL)	101.0 ± 12.7	94.0 ± 11.6***
Insulin (μUI/mL)	21.5 ± 17.1	14.1 ± 8.0***
HOMA-IR	5.5 ± 4.8	3.4 ± 2.7***
A1c (%)	5.6 ± 0.4	5.4 ± 0.4***
TC (mg/dL)	187.8 ± 33.4	178.2 ± 44.2*
HDL-C (mg/dL)	45.9 ± 9.9	46.7 ± 11.2
LDL-C (mg/dL)	115.3 ± 25.4	111.8 ± 36.2
TG (mg/dL)	118.0 (92.8; 157.0)	93.5 (72.0; 125.5)**
Uric acid (mg/dL)	6.0 ± 1.6	5.5 ± 1.6 ***

Data are presented as mean ± SD for parametric data and median (IQ range) for non-parametric parameters. *p < 0.05; **p < 0.01; ***p < 0.001 when compared with a paired Student *t*-test or U Mann–Whitney (parametric and non-parametric parameters, respectively).

BMI, body mass index; BP, blood pressure; HOMA-IR, insulin resistance index; A1c, hemoglobin A1c; TC, total cholesterol; HDL-C, high-density lipoprotein cholesterol; LDL-C, low-density lipoprotein cholesterol; TG, triglycerides.

**Table 2:** Inflammatory and hematologic parameters outcomes in the study population.

Parameter	Baseline	Final
hs-CRP (g/dL)	7.6 (4.3; 11.1)	4.9 (2.6; 11.2)**
C3 Protein (mg/dL)	138.5 ± 20.2	126.3 ± 19.2***
RBP4 (mg/dL)	2.1 ± 0.4	1.9 ± 0.6
Leukocyte count (×10^9^/L)	7.9 ± 2.0	7.4 ± 2.1*
Neutrophil count (×10^9^/L)	4.6 ± 1.4	4.3 ± 1.5*
Lymphocyte count (×10^9^/L)	2.5 ± 0.8	2.4 ± 0.9
Monocyte count (×10^9^/L)	0.58 ± 0.16	0.54 ± 0.17*
Eosinophil count (×10^9^/L)	0.22 ± 0.16	0.19 ± 0.13
Basophils count (×10^9^/L)	0.04 ± 0.05	0.04 ± 0.05
SII (×10^9^/L)	523.5 ± 234.6	459.1 ± 220.2**
Adiponectin (pg/mL)	2254519.4 ± 13920052.9	28746386.5 ± 25114908.8**
PAI1 (pg/mL)	131841.3 ± 61172.3	101650.9 ± 36920.0***

Data are presented as mean ± SD for parametric data and median (IQ range) for non-parametric parameters. *p < 0.05; **p < 0.01; ***p < 0.001 when compared with a paired Student *t*-test or U Mann–Whitney (parametric and non-parametric parameters, respectively).

C3, complement component 3; hs-CRP, high-sensitivity C-reactive protein; PAI1, Plasminogen activator inhibitor 1; RBP4, Retinol Binding Protein 4; SII, systemic immuneinflammation index (SII = platelet × neutrophil/lymphocyte ratio).

### 3.1 Quality of sleep

**Figure 1:**
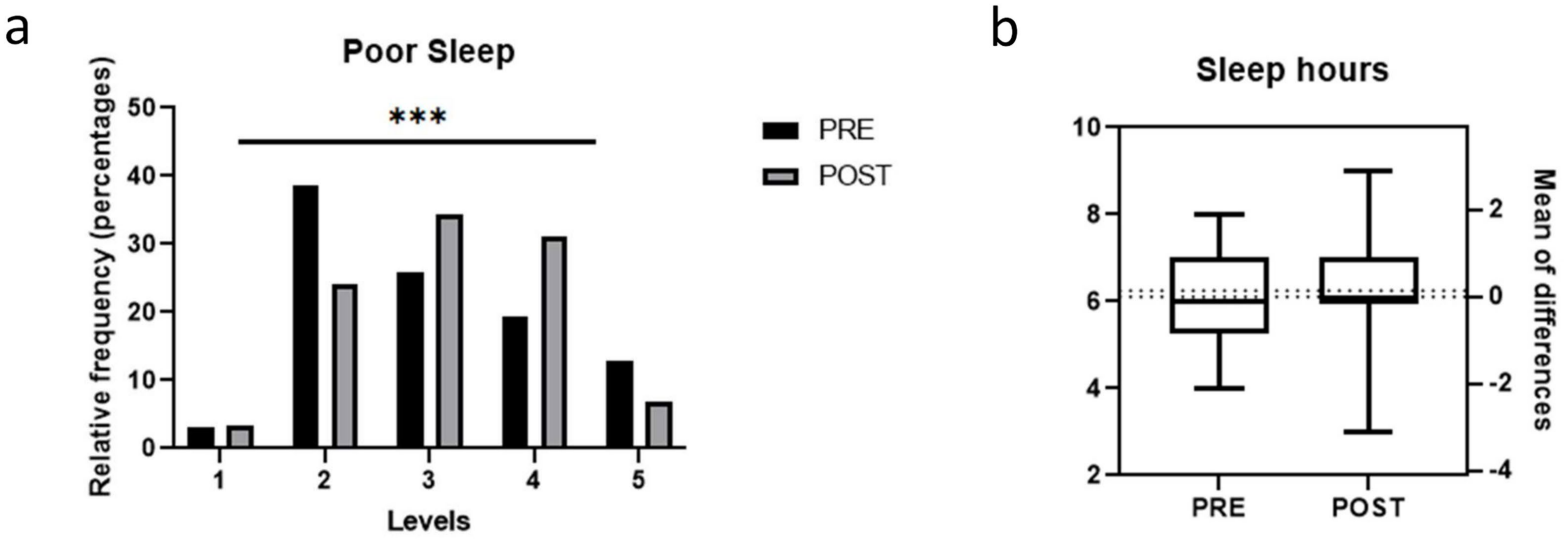
Quality of sleep in the study population before and after the weight loss intervention. **(a)** Frequency of poor sleep (1 Always, 2 Often, 3 Sometimes, 4 Rarely, 5 Never); **(b)** Hours of sleep. Bars represent proportions and pre-post comparisons were analyzed by chi-square test.

After completing the six-month dietary intervention, significant improvements were observed in the participants’ sleep quality, with a significant reduction in the number of nights of poor sleep (*χ*^2^ = 60.7; *p* < 0.001) (Figure 1a). However, no significant changes were observed in total hours slept after the intervention (Figure 1b).

### 3.2 Self-esteem

**Figure 2:**
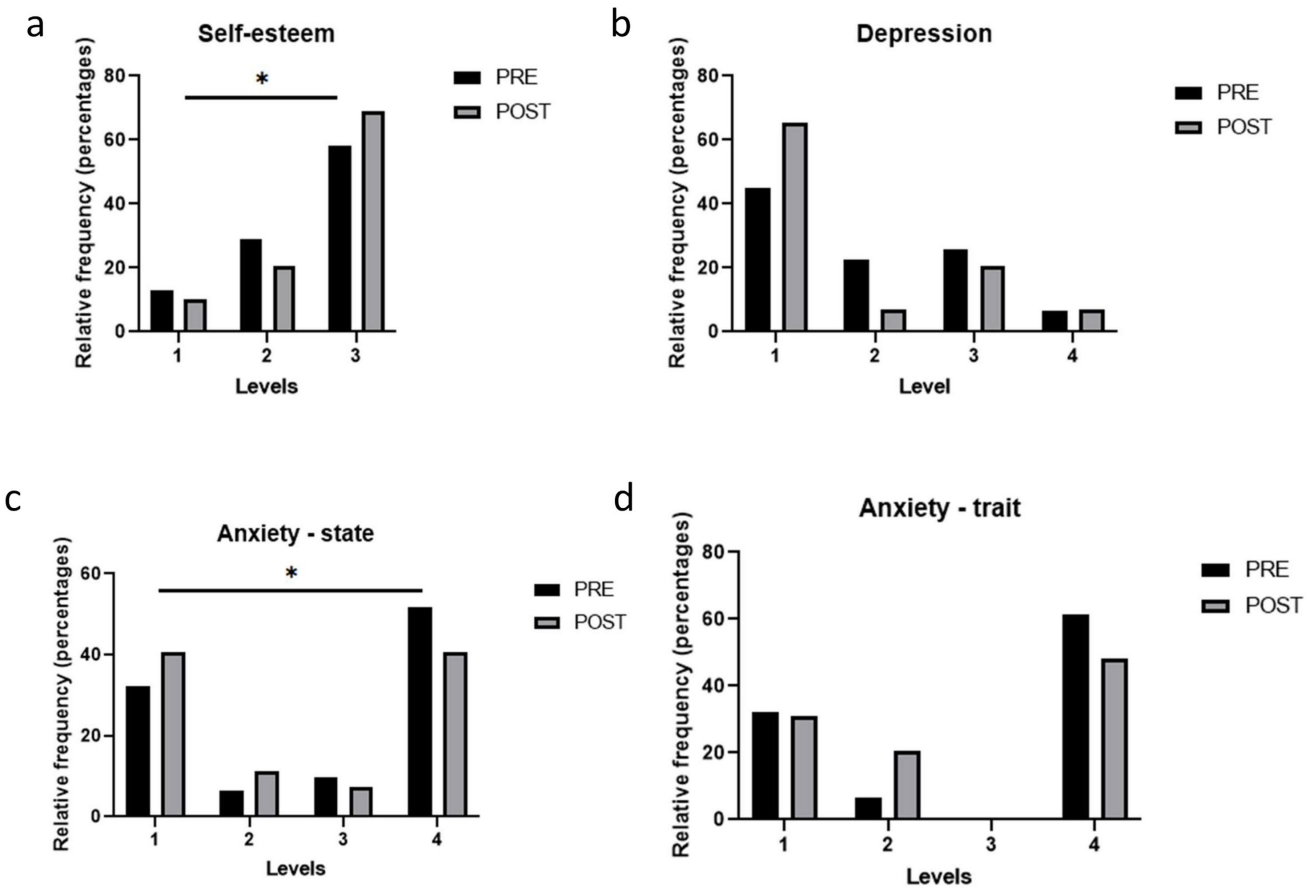
Psychological issues in the study population before and after the weight loss intervention. **(a)** Levels of Self-Esteem according to the Rosenberg Self-Esteem Scale (1 Low; 2 Moderate; 3 High); **(b)** Levels of Depression according to the Beck Depression Inventory (BDI-II) (1 Minimal; 2 Mild; 3 Moderate, 4 Severe); **(c,d)** State and Trait Anxiety according to the State-Trait Anxiety Inventory (STAI) (1 Low; 2 Moderate; 3 High). Bars represent proportions and pre-post comparisons were analyzed by chi-square test.

Analysis of the total scores on the Rosenberg Self-Esteem Scale showed no significant overall improvement in self-esteem following the intervention (pre vs. post: 31.7 ± 5.4 vs. 32.4 ± 5.3; *p* = 0.35). However, analysis of self-esteem categories revealed a clear shift, with a marked increase in the proportion of subjects classified as having high self-esteem after the dietary intervention (pre vs. post: 62% vs. 69%; *χ*^2^ = 12.5; *p* < 0.05) (Figure 2a).

### 3.3 Depression

The BDI-II was used to assess depression before and after the intervention. Minimal depression cases increased, while mild and moderate decreased, but changes were not statistically significant (Chi-square, *p* = 0.122) (Figure 2b).

### 3.4 State and trait anxiety

Regarding the total score, state anxiety improved after the 6-month intervention (STAI-S pre vs. post: 22.6 ± 12.9 vs. 17.9 ± 10.8; *p* < 0.01), whereas no statistically significant change occurred with respect to trait anxiety (STAI-T pre vs. post: 24.2 ± 12.8 vs. 22.4 ± 11.7; *p* = 0.32). At the categorical level, there were significant changes in state anxiety after the intervention, characterized primarily by an increase in the proportion of subjects with very low anxiety and a decrease in those with high anxiety (Figure 2c). In contrast, although the differences in trait anxiety categories did not reach statistical significance (*p* = 0.089), a trend towards a decrease was observed in the proportion of subjects with moderate and high levels of anxiety (Figure 2d).

### 3.5 Circadian gene expression levels in PBMCs

**Figure 3:**
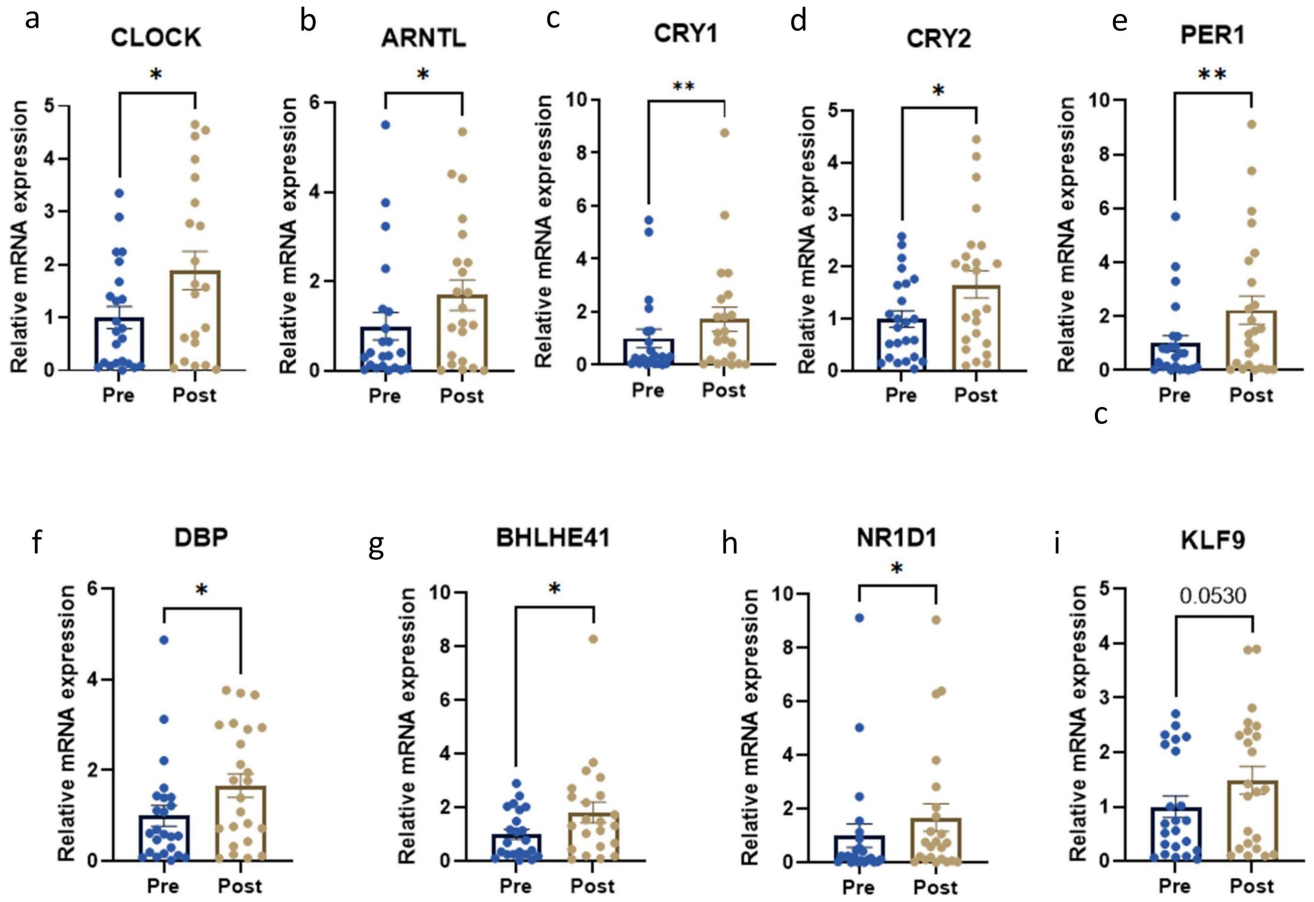
Circadian gene expression levels before and after the weight loss intervention. *p < 0.05; **p < 0.01; ***p < 0.001 when compared with a paired Student *t*-test. Relative mRNA expression of **(a)** CLOCK; **(b)** ARNTL; **(c)** CRY1; **(d)** CRY2; **(e)** PER1; **(f)** DBP; **(g)** BHLHE41; **(h)** NR1D1; **(i)** KLF9.

We evaluated the expression of key circadian genes in subjects’ PBMCs before and after the dietary intervention. Interestingly, core clock genes were significantly up regulated, namely *CLOCK* (*p* < 0.05), *ARNTL* (*p* < 0.05), *CRY1* (*p* < 0.01), *CRY2* (*p* < 0.05) and *PER1* (*p* < 0.01) (Figures 3a–e). In addition, the expression levels of the secondary circadian regulated genes were also significantly increased, in particular *DBP* (*p* < 0.05), *BHLHE41* (*p* < 0.05) and *NR1D1* (*p* < 0.05) (Figures 3f–h). In contrast, no statistically significant changes were observed for *KLF9* after the intervention (*p* = 0.05) (Figure 3i).

### 3.6 Association between circadian gene expression and psychological variables

The results at baseline indicated a significant association between *CLOCK* and state anxiety (*χ*^2^ = 20.020, *p* < 0.05), suggesting individuals with higher *CLOCK* expression levels were more likely to have lower levels of state anxiety. In addition, *CRY2* was associated with self-esteem and depression (*χ*^2^ = 6.964 and *χ*^2^ = 18.333, *p* < 0.05 respectively). Thus, individuals with higher *CRY2* levels were more likely to have higher self-esteem and lower levels of depressive symptoms. At the end of the intervention these associations remained significant, in addition to higher *CRY1* expression alongside lower state anxiety symptoms (*χ*^2^ = 8.071, *p* < 0.05).

### 3.7 Correlation analysis of circadian gene expression and inflammation-related parameters

**Figure 4:**
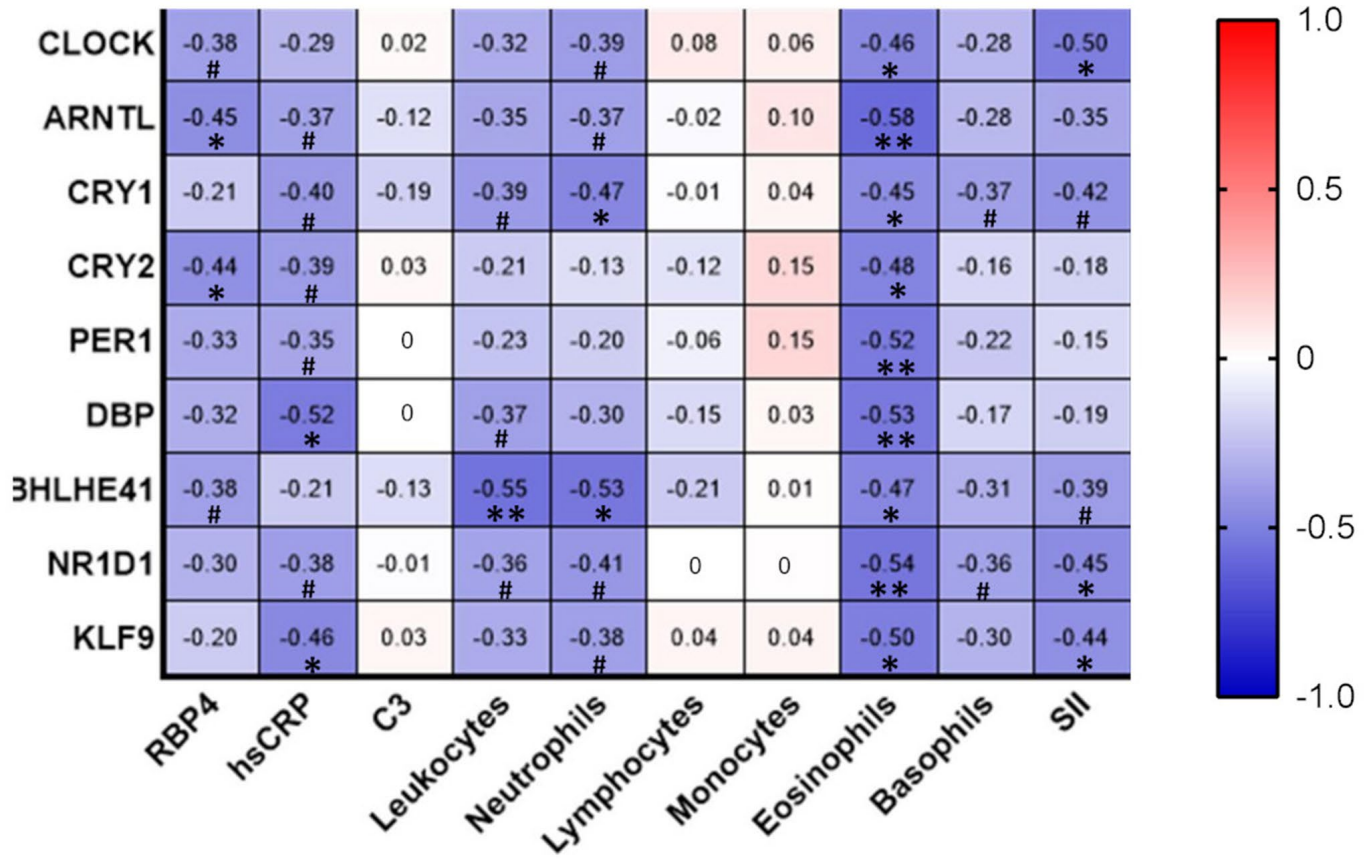
Correlation analysis of circadian gene expression and inflammation-related parameters. Correlations between variables were analyzed using Spearman’s Rho bivariate correlation. *p < 0.05; **p < 0.01;·# between *p* > 0.05 and *p* < 0.10. C3, complement component 3; hs-CRP, high-sensitivity C-reactive protein; RBP4, Retinol Binding Protein 4; SII, systemic immune-inflammation index (SII = platelet × neutrophil/lymphocyte ratio).

Interestingly, the expression of many of the circadian genes correlated negatively with inflammatory markers. Specifically, we observed that retinol binding protein 4 (RBP4) negatively correlated with *CRY2* (*r* = −0.44, *p* < 0.05) and *ARNTL* (*r* = −0.45, p < 0.05). hsCRP levels negatively correlated with *DBP* (*r* = −0.52, p < 0.05) and *KLF9* (*r* = −0.46, p < 0.05). Leukocyte count negatively correlated with *BHLHE41* (*r* = −0.55, *p* < 0.01). The number of neutrophils was also negatively associated with circadian marker *CRY1* (*r* = −0.47, *p* < 0.05) and *BHLHE41* (*r* = −0.53, *p* < 0.05). Eosinophil count correlated negatively with all the genes analyzed. Finally, SII, which estimates the systemic immune-inflammatory response, negatively correlated with *CLOCK* (*r* = −0.50, *p* < 0.05), *NR1D1* (*r* = −0.45, *p* < 0.05) and *KLF9* (*r* = −0.44, *p* < 0.05). Finally, although not reaching statistical significance, RBP4, hsCRP, leukocyte count, neutrophil number, and SII showed a tendency to negatively correlate with several circadian-related genes, as indicated in Figure 4.

## 4 Discussion

This study explores the relationship between weight loss, circadian gene expression regulation, and emotional well-being in the context of obesity. Our results show that subjects who followed a six-month dietary intervention experienced a significant reduction in body weight, accompanied by improvements in metabolic parameters and a widespread decrease in inflammatory markers. At the molecular level, an upregulation of key circadian clock genes was evident in PBMCs, and was associated with improved psychological and metabolic markers. Furthermore, significant negative correlations were identified between the expression of circadian genes and various inflammatory markers, suggesting that diet modulates not only metabolism and emotional state but also the expression of molecular clock related genes and systemic inflammation. These findings underscore the importance of a holistic approach to addressing the complex interplay between metabolic and psychological factors in obesity management.

Following the weight loss intervention, subjects with obesity showed a significant improvement in several psychological dimensions, including self-esteem, depression, and anxiety, with the most pronounced change being observed in state anxiety. These findings are in accordance with a meta-analysis by Jones et al. (15), who reported enhanced self-esteem and body image satisfaction in adults who had achieved a 5%–10% reduction in their body weight. In addition, our study reveals a novel association between the circadian gene *CRY2* and self-esteem, suggesting for the first time that higher *CRY2* expression correlates with improved self-esteem, a relationship not previously documented in the literature. In terms of depression, prior research has shown that combined dietary and physical activity interventions lead to significant decreases in depressive symptoms (16). Such improvements in depressive symptoms may result from both physiological changes and increased psychological factors, such as a sense of control and accomplishment (17). In this context, altered circadian gene expression -particularly that involving *CRY2* - has been linked to depression and sleep disorders, reinforcing the connection between circadian rhythm disruption and mood disturbances (18).

Regarding anxiety outcomes, weight loss interventions have been associated with reductions in both state (situational) and trait (predisposition) anxiety (19). Our data show a significant decrease in state anxiety, consistent with previous studies in which physical activity and improved body image contributed to decreased anxiety symptoms over time. Additionally in those studies, trait anxiety improved over time in some subjects, probably due to increased physical activity and reduced body dissatisfaction. In addition, due to cross-communication between neurohormones, inflammatory pathways, and circadian gene expression, psychological disorders are closely related to each other; specifically, the *CLOCK* gene can directly affect anxiety symptoms. Satyanarayanan et al. (20) observed that reduced expression of the *CLOCK* gene is associated with higher levels of generalized anxiety, in accordance with our present results. In our study, we observed a higher expression of the *CRY1* gene in subjects with low levels of state anxiety. This finding contrasts with previous reports that have associated elevated expression of *CRY1* and *CRY2* with higher anxiety levels in both women and men (21). Nonetheless, despite these discrepancies, the literature consistently reports a strong link between circadian rhythm deregulation and the clinical manifestation of psychological disorders, underscoring the bidirectional nature of this relationship (11, 18, 20, 22). In this sense, some studies have suggested that addressing sleep disorders should be a crucial target of interventions for depression and anxiety, in order to contribute to the restoration of normal circadian rhythms (22).

Quality of sleep plays a fundamental role in both metabolic regulation and psychological well-being. Poor sleep alters the hormones that control appetite; namely, there is a reduction in leptin and an increase in ghrelin, which can influence body weight (23). In turn, insufficient sleep can affect mental and emotional functioning (24). In our subjects, although total sleep duration remained unchanged, the frequency of nights with poor sleep decreased significantly after the intervention. This highlights the direct impact that improving sleep has on psychological health and on eating behaviors that contribute to obesity (25).

Mounting evidence underscores the relevance of circadian gene expression in immune cells and supports the use of PBMCs as reliable markers of circadian rhythm (26–28). Remarkably, Wittenbrink et al. (28) developed the BodyTime assay, using circadian genes in human monocytes to reflect the master clock. These genes showed consistent circadian oscillations, and BodyTime correlated strongly with dim light melatonin onset, the gold standard for internal circadian phase. Our data reveals that a 6-month calorie restriction intervention in subjects with obesity has profound effects on circadian gene expression in PBMCs by consistently increasing their expression. To our knowledge, this is the first study to examine how a structured VLCD intervention modulates the expression of circadian-related genes in PBMCs. We have previously shown that PBMC circadian-related core clock proteins, such as *CLOCK*, phosphorylated *BMAL1*, *PER2* and *CRY1* (among others), are diminished in overweight subjects with type 2 diabetes, and that these alterations are associated with increased inflammatory parameters and enhanced leukocyte-endothelial cell interactions (29). Obesity is related with enhanced subclinical atherosclerosis, manifested as decreased leukocyte rolling velocity and enhanced rolling flux and adhesion, which have been shown to improve when a dietary weight loss intervention based on VLCD and LCD is implemented (30). In the present study, along with increases in circadian gene expression levels, a decrease in inflammatory markers, such as hs-CRP, C3 protein, neutrophil and monocyte count, was observed after the dietary intervention, which together are likely to contribute to improvements in subclinical atherosclerosis.

Many studies have shown that circadian alignment of food intake – i.e., restricting meals to a specific time window that matches the active phase of the individual - has multiple benefits, including a reduction of body weight and an improvement in glucose-related parameters and cardiometabolic risk factors in subjects with obesity (31). However, these benefits are not superior to those afforded by other dietary interventions, such as daily calorie restriction (32). In addition, the timing of meals in some of studies reporting metabolic benefits might be difficult to apply to normal daily life, as they included prolonged fasting periods in the active phase of the day (33). Therefore, calorie restriction VLCDs continue to be the most effective short-term strategy for reducing weight and metabolic syndrome characteristics (34).

It is known that calorie restriction modulates circadian rhythm; indeed, the underlying mechanisms have been characterized in different species and would seem to be evolutionarily preserved (35). Metabolic adaptation to calorie restriction involves modulation of circadian gene expression, as demonstrated by changes in circadian gene expression in the liver of mice (36). Furthermore, calorie restriction induces a shift from 24-h rhythms in metabolism-related gene expression to 12-h rhythms in mice under calorie restriction, changes that correlate with improvements in glucose homeostasis (37). Our study is pioneer in highlighting that peripheral blood cells also undergo important gene expression changes after a period of calorie restriction. These cells are known to incorporate rhythmic expression of circadian and inflammatory genes, (38) and, interestingly, can be modified by diet. Indeed, switching from a high-carbohydrate, low-fat diet to a low-carbohydrate, high fat diet has been shown to alter diurnal oscillations of core clock genes in monocytes (39).

Feeding-fasting cycles regulate peripheral circadian rhythms in metabolic organs, as food intake is a strong zeitgeber that controls the molecular clocks of liver and adipose tissue, which are disrupted in obesity and type 2 diabetes (40). Daily oscillations in intestinal microbial communities can regulate the circadian transcriptional program in metabolic tissues (41). The gut microbiome emerges as a central modulator of obesity, affecting energy storage and expenditure systemic inflammation and metabolic endotoxemia (42). Importantly, alterations in gut microbiota composition are also associated with psychiatric conditions, such as eating disorders, anxiety, depression and low self-esteem, which often coexist with obesity and exacerbate systemic inflammation and metabolic dysfunction (43). Recent evidence shows that a multiphase dietary intervention in obese individuals induces positive changes in gut microbiota composition, particularly increasing the abundance of beneficial taxa in metabolically unhealthy participants, demonstrating that dietary induced modulation of the microbiome can improve both metabolic and inflammatory profiles (44). Taken together, these findings suggest that targeting the gut microbiome could modulate both metabolic and psychological outcomes, highlighting its potential as a key focus for future interventions.

Therefore, a deeper understanding of the complex interactions between circadian rhythms, metabolic tissues, obesity development and psychological factors is essential. This comprehensive knowledge would enable a multidisciplinary approach to tackling obesity, leading to more precise and effective interventions tailored to address its multifaceted nature (45).

The present study has several limitations that should be acknowledged. First, the lack of determination of oscillations in circadian gene expression, as well as protein expression limits the understanding of potential biological mechanisms. Although all blood samples were collected at standardized times, residual variability related to chronotype, sleep–wake cycle, and food timing cannot be fully excluded. In addition, adherence to the very low-calorie diet may have varied among participants despite regular follow-up and monitoring. We acknowledge that validated questionnaires (e.g., PSQI) could provide more standardized assessments for sleep quality. Furthermore, the study’s focus on a very specific group (middle-aged individuals with obesity) restricts its generalizability. However, our group has previously shown that overweight individuals with type 2 diabetes exhibit altered circadian gene expression in PBMCs (29), underscoring that metabolic impairment can significantly impact the expression of these genes. Although the lack of a control group limits causal inference, observed psychological improvements may reflect placebo or psychosocial effects, yet the study still offers valuable insights into responses to a structured multiphase dietary intervention (46). Finally, we acknowledge that our findings linking circadian markers and psychological outcomes are correlational in nature. Future intervention studies targeting circadian rhythms — e.g. by time-restricted eating — will be essential to establish causal relationships and further elucidate the mechanisms underlying these associations.

One of the strengths of the study is its comprehensive approach to addressing a weight loss intervention in obesity, which goes beyond focusing solely on metabolic aspects. By incorporating emotional and psychological dimensions, our work provides a broader understanding of the complex problems associated with obesity. Additionally, the analysis of circadian gene expression in relation to emotional state is a significant strength, as it offers a valuable biological perspective that could pave the way for innovative therapeutic solutions to psychological disorders linked to obesity. In this way, ours multifaceted approach enriches the field by integrating biological, emotional, and psychological insight.

In summary, although exploratory, our findings highlight the interplay between weight loss, circadian gene expression regulation and emotional well-being in people with obesity. Weight reduction not only improves metabolic health, but also affords positive psychological outcomes, including reductions in anxiety and depression, along with an improvement in self-esteem. It is clear that circadian gene expression, particularly in the *CRY2* gene, plays a relevant role in the modulation of psychological variables. However, the relationship between circadian rhythms and mental health is complex and requires further research to fully understand the underlying mechanisms. Overall, our study highlights the need for holistic approaches that integrate changes in lifestyle, diet, and sleep regulation to improve both the physical and emotional health of people with obesity.

## Data Availability

The raw data supporting the conclusions of this article will be made available by the authors, without undue reservation.
