# Supplementary material for: Weight loss increases circadian gene expression and emotional well-being in individuals with obesity
Source: Front Nutr. 2025 Nov 26;12:1722428. doi: 10.3389/fnut.2025.1722428 (PMC12689339; doi:10.3389/fnut.2025.1722428)
Supplement: Supplementary file 1 [file Table_1.docx]

Supplementary Material

# Supplementary Table 1. Sequences of the primers used for RT-qPCR.

| Gene | Direction | Sequence |
| --- | --- | --- |
| *CLOCK* | Forward | CAGGCAGCATTTACCAGCTCATG |
|  | Reverse | GTAGCTTGAGACATCACTGGCTG |
| *ARNTL* | Forward | GCTCAGGAGAACCCAGGTTATC |
|  | Reverse | GCATCTGCTTCCAAGAGGCTCA |
| *CRY1* | Forward | GCAGTTGCTTGCTTCCTGACAC |
|  | Reverse | GACAGCCACATCCAACTTCCAG |
| *CRY2* | Forward | AGGAGAACCACGACGAGACCTA |
|  | Reverse | CCGTTCCAAGTGCTTATCCAGG |
| *PER1* | Forward | TCAACTGCCTGGACAGCATCCT |
|  | Reverse | TCAGAGGCTGAGGAGGTGGTAT |
| *DBP* | Forward | CCTTTGACCCTCGAAGACATCG |
|  | Reverse | TGCCTCGTTGTTCTTGTACCGC |
| *BHLHE41* | Forward | CTGGGACATCTGGAGAAAGCTG |
|  | Reverse | AGTGGAACGCATCCAAGTCGGA |
| *NR1D1* | Forward | CTGCCAGCAATGTCGCTTCAAG |
|  | Reverse | TGGCTGCTCAACTGGTTGTTGG |
| *KLF9* | Forward | CTACAGTGGCTGTGGGAAAGTC |
|  | Reverse | CTCGTCTGAGCGGGAGAACTTT |
| *18S* | Forward | ACCCGTTGAACCCCATTCGTGA |
|  | Reverse | GCCTCACTAAACCATCCAATCGG |
